# Draft genome sequence of *Enterobacter cloacae* ST473 harbouring *bla*CMH-3 isolated from a human patient diagnosed with recurrent bacteriuria in Nigeria

**DOI:** 10.1099/acmi.0.000565.v3

**Published:** 2023-07-06

**Authors:** Ebuka Elijah David, Ikechuku Okorie Igwenyi, Ifeanyichukwu Romanus Iroha, Layla Farage Martins, Guillermo Uceda-Campos, Aline Maria da Silva

**Affiliations:** ^1^​ Department of Biochemistry, Alex Ekwueme Federal University, Ndufu-Alike, Ikwo, Nigeria; ^2^​ Department of Biochemistry, Institute of Chemistry, University of Sao Paulo, Sao Paulo, Brazil; ^3^​ Department of Biochemistry, Ebonyi State University, Abakaliki, Ebonyi State, Nigeria; ^4^​ Department of Microbiology, Ebonyi State University, Abakaliki, Ebonyi State, Nigeria

**Keywords:** *Enterobacter cloacae*, extended-spectrum beta-lactamases, urinary tract infection

## Abstract

*
Enterobacter cloacae* is among the most frequently isolated species described in clinical infections and is commonly associated with a multidrug resistance (MDR) phenotype. We present the draft genome sequence of a MDR *E. cloacae* isolated in Nigeria from the urine sample of an adult male outpatient diagnosed with symptomatic recurrent bacteriuria. The isolate was found to be resistant to ceftriaxone, cefotaxime, cefepime and levofloxacin. Genome analysis revealed the presence of the beta-lactamase chromosomal gene *bla*CMH-3, which may be responsible for the antibiotic resistance observed in the recurrent *E. cloacae* urinary tract infection.

## Data Summary

The first version of draft genome assembly has been deposited in DDBJ/ENA/GenBank under the accession number JAPOMC000000000 (BioProject accession number PRJNA864081 and BioSample accession number SAMN30073201). The raw sequence reads have been deposited in the Sequence Read Archive (SRA) under the accession number SRR20725478. The 16S rRNA sequence was deposited with GenBank accession number OP932952. Modified double disc synergy tests showing synergy of the zone of inhibition between amoxicillin-clavulanic acid (AMC) and the cephalosporins are shown in the Supplementary Material.

## Announcement

*Enterobacter cloacae* complex is among the *Enterobacter* species most frequently described in clinical infections, particularly in immune-compromised patients and those hospitalized in intensive care units. *Enterobacter* spp. clinical isolates are commonly associated with a multidrug resistance (MDR) phenotype, which makes their treatment challenging. Isolates from *Enterobacter* species are reported to present intrinsic resistance to ampicillin, amoxicillin-clavulanic acid, first- and second-generation cephalosporins due to the chromosomal encoded AmpC beta-lactamase [1]. Furthermore extended-spectrum beta-lactamases (ESBL) have also been reported in *Enterobacter* species [1, 2]. Analysis of 272 *E. cloacae* genome assemblies belonging to a variety of STs showed that almost all of them encode a β-lactamase gene with *bla*CMH and *bla*ACT being the main *bla*AmpC genes [2]. Among the *bla*CMH genes found in this 272-genome dataset, *bla*CMH-6 (*n*=41), *bla*CMH-4 (*n*=26), *bla*CMH-5 (*n*=21), *bla*CMH-3 (*n*=21) and *bla*CMH-1 (*n*=16) were the most prevalent [2]. *bla*CMH-1 has been identified as plasmid-mediated β-lactamase gene in *E. cloacae* clinical isolates collected in Southern Taiwan in 2015 [3]. *bla*CMH-3 was identified as a chromosome encoded gene in a *E. cloacae* clinical isolate assigned to ST932 harbouring a plasmid-mediated *bla*NDM-1, which was collected in Spainin 2016 from a Ukraine patient [4]. To our knowledge, no other clinical cases of *E. cloacae* harbouring *bla*CMH-3 has been reported despite the detection of this *bla*AmpC variant in several publicly available *E. cloacae* genomes [2]. Here we report the draft genome sequence of an *E. cloacae* isolate collected in March 2021 at the Laboratory unit of Alex Ekwueme Federal Teaching Hospital Abakaliki, Ebonyi State, Nigeria from the urine sample of an adult male outpatient with recurrent urinary tract infection (UTI).

The isolate was collected, stored in nutrient broth with glycerol. Antibiotic susceptibility testing was performed by the disk diffusion method according to the Clinical and Laboratory Standards Institute (CLSI) guidelines 2021 on Muller–Hinton (MH) agar [5]. After 24 h of growth at 37 °C, the isolate was evaluated for its susceptibility to ciprofloxacin (5 µg), levofloxacin (5 µg), cefepime (30 µg), ceftriaxone (30 µg), cefotaxime (30 µg), ceftazidime (30 µg), amoxicillin-clavulanic acid (20/10 µg), ampicillin (10 µg), imipenem (10 µg) and fosfomycin (200 µg). A modified DDST (modified double disc synergy test) with the use of cefepime was also performed to improve the detection of ESBL-producing isolates, which co-produce AmpC [6] (Fig. S1, available in the online version of this article). The isolate was found to be resistant to the two third generation cephalosporins (ceftriaxone and cefotaxime) and showed intermediate resistance (MIC of 15 µg ml^−1^ with zone of inhibition of 18.4 mm ±0.01) to cefepime, a fourth-generation cephalosporin. The isolate was susceptible to amoxicillin-clavulanic acid and imipenem, and resistant to ampicillin. Regarding the fluoroquinolones, the isolate showed intermediate resistance to ciprofloxacin (MIC of 0.5 µg ml^−1^ with zone of inhibition of 23 mm ±0.00) and resistance to levofloxacin. We also verified that the isolate showed resistance to fosfomycin.

The isolate was cultured in Tryptic Soy Agar (TSB) broth and 0.5 ml was used for genomic DNA extraction with the Wizard Genomic Purification kit (Promega, USA). Purified DNA was used as a template for 16S rRNA gene amplification with universal primers 27F (5ʹAGAGTTTGATCMTGGCTCAG-3ʹ) and 1525R (5'- AAGGAGGTGWTCCARCCGCA-3′) as previously described [7]. The amplicon was subjected to sequencing reactions using Big Dye terminator v3.1 cycle sequencing kit (ThermoFisher Scientific) with 27F or 1525R as sequencing primers and then injected on an ABI PRISM 3130XL genetic analyser (ThermoFisher Scientific). The obtained sequences were used to assemble a 16S RNA 1546 bp sequence, which was then blastn searched against the GenBank/NCBI database allowing taxonomic identification of the clinical isolate as *E. cloacae* (100 % identity, 100 % query cover).

Whole-genome sequencing of the *E. cloacae* isolate was performed on Illumina MiSeq platform (Illumina, CA, USA) at the Center for Advanced Technologies in Genomics (CATG), Institute of Chemistry, University of Sao Paulo, using the genomic DNA purified with Wizard Genomic Purification kit (Promega, USA) DNA. The shotgun genomic library was prepared using the Nextera DNA Library Prep (Illumina) with a total DNA input of ~20 ng and subjected to a run using an Illumina MiSeq Reagent Kit v3 (2×300 cycles). The reads were checked for quality phred=30 and filtered using Fastp v0.12.4 [8]. Filtered rawR1 reads were used for *de novo* assembly using SPAdes v3.15.4 [9]. Assembly completeness and contamination were verified with CheckM v. v1.2.0 [10] and the assembly quality was evaluated with quast v5.0.2 [11]. The assembled genome was annotated using prokka v.1.14.6 [12]. General features of the genome assembly and annotation are presented in Table 1.

**Table 1.** General features of the *E. cloacae*draft genome

Assembly							Annotation		
Contigs number	Estimated chromosome length (bp)	N50 (bp)	GC (%)	Completeness	Contamination	Sequencing coverage	CDSs	tRNAs	rRNA operons
15	5 012 585	857 935	55.12	99.97	0.33	125 x	4657	72	8

Sequence type was defined using MLST v 2.0.9 and multi locus sequence typing (MLST) allele sequences and profile data obtained from PubMLST.org [13, 14]. ResFinderFG v 2.0 was used to identify antibiotic resistance determinants [15]. MobileElementFinder v 1.0.3 and ISfinder were used to identify insertion sequences and mobile genetic elements and their relation to antimicrobial resistance genes and virulence factors [16, 17]. Table 2 lists the antibiotics resistance genes (ARGs) detected in the *E. cloacae* draft genome. It is worth noting a chromosomal *blaCMH-3* gene with 100 % identity and 100 % coverage to the reference gene. Hence, this is the first time multidrug-resistant *E. cloacae* with an ESBL phenotype harbouring a single narrow spectrum *blaCMH-3* beta-lactamase is reported in Nigeria. A fosfomycin resistance gene *fosA* was also detected. Genes of the RND (resistance nodulation division) drug efflux system, *OqxAB*, related to fluoroquinolone resistance was also observed. The *E. cloacae* isolate seems to carry a plasmid without antibiotic resistance genes, which is similar to plasmid ColRNAI originally found in *Klebsiella pneumoniae* strains where it encodes carbapenems resistance genes [18]. The genomic features of *E. cloacae* clinical isolate described here suggest that a single AmpC beta-lactamase may be responsible for a broader extended spectrum beta-lactamase drug resistance. Despite the cost associated to whole-genome sequencing of clinical isolates, which might be prohibitive in regions with limited resources, elucidating the genetic basis of resistance is highly valuable to enable initial antibiotic selection to minimize use of ineffective antibiotics [19]. Based solely on the susceptibility testing phenotypic assays, a successful treatment of the recurrent UTI reported in this study could be achieved with amoxicillin-clavulanic acid. However, in vitro susceptibility does not necessarily translate to in vivo susceptibility. This may be related to the current clinical practice of high amoxicillin-to-clavulanate ratios, which has been found to result in the most rapid adaptation to antibiotics via gene dosing responses [20].

**Table 2.** Antibiotic resistance genes detected in *E. cloacae* genome assembly

Gene	Description	Protein identity %	Query coverage %	Reference accession no.
*bla*CMH-3	Cephalosporin-hydrolysing class C beta-lactamase CMH-3	100	100	WP_045295573.1
*fos*A	fosfomycin resistance glutathione transferaseFosA	100	100	AFM58300.1
OqxAB	multidrug efflux RND permease	100	100	WP_262761476.1

## Supplementary Data

Supplementary material 1
