# Supplementary material for: Draft genome sequence of Enterobacter cloacae ST473 harbouring blaCMH-3 isolated from a human patient diagnosed with recurrent bacteriuria in Nigeria
Source: Access Microbiol. 2023 Jul 6;5(7):acmi000565.v3. doi: 10.1099/acmi.0.000565.v3 (PMC10436016; doi:10.1099/acmi.0.000565.v3)
Supplement: Supplementary material 1 [file acmi-5-565.v3-s001.pdf]

Supplementary file

Draft genome sequence of *Enterobacter cloacae* ST473 harbouring *bla*CMH-3 isolated from a human patient diagnosed with recurrent bacteriuria in Nigeria

Ebuka Elijah David[1,4*], Ikechuku Okorie Igwenyi[2], Ifeanyichukwu Romanus Iroha[3], Layla Farage Martins[4], Guillermo Uceda-Campos[4]and Aline Maria da Silva[4]

Author affiliations:

[1]Department of Biochemistry, Alex Ekwueme Federal University, Ndufu-Alike, Ikwo, Nigeria

[2]Department of Biochemistry, Ebonyi State University, Ebonyi State, Nigeria

[3]Department of Microbiology, Ebonyi State University, Ebonyi State, Nigeria

[4]Department of Biochemistry, Institute of Chemistry, University of Sao Paulo, Brazil

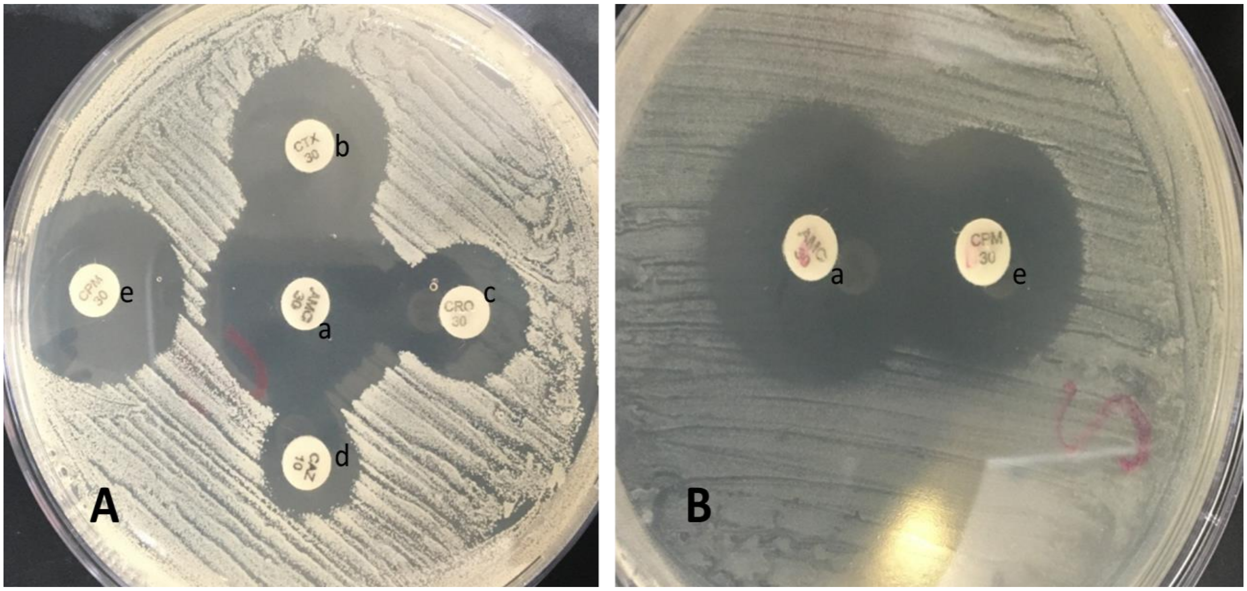

FigureS1: Modified Double Disc Synergy Test (DDST) showing synergy of the zone of inhibition between amoxicillin-clavulanic acid (AMC), **a**, and third generation of cephalosporins (cefotaxime, **b**, ceftriaxone, **c,**ceftazidime, **d**) (shown in panel **A**) and fourth generation of cephalosporins (cefepime, **e**) (shown in panel **B**). Synergy between the zone of inhibitions of **a** and **b, c, d** indicates phenotypically that the isolate is ESBL-producing. Synergy between the zone of inhibitions of **a** and **e** indicates phenotypically that the isolate is AmpC-producing.
